# Draft Genome Sequence of *Lutibaculum baratangense* Strain AMV1^T^, Isolated from a Mud Volcano in Andamans, India

**DOI:** 10.1128/genomeA.00735-14

**Published:** 2014-07-24

**Authors:** Aditya Singh, Ara Sreenivas, Gundlapally Sathyanarayana Reddy, Anil Kumar Pinnaka, Sisinthy Shivaji

**Affiliations:** a CSIR-Institute of Microbial Technology, Chandigarh, India; b CSIR-Centre for Cellular and Molecular Biology, Hyderabad, Andhra Pradesh, India

## Abstract

The 4.3-Mb genome of *Lutibaculum baratangense* strain AMV1^T^, isolated from a soil sample collected from a mud volcano in Andamans, India, is reported. The draft genome of strain *Lutibaculum baratangense* AMV1^T^ consists of 4,300,776 bp with a G+C content of 66.93 mol% and 4,198 predicted coding regions, including 56 RNAs.

## GENOME ANNOUNCEMENT

*Lutibaculum baratangense* AMV1^T^ is a Gram-staining negative, motile, oval-to-rod shaped bacterium isolated from a soil sample of a mud volcano in Baratang Island, Andamans, India (1). Because of their similarity to 16S rRNA gene sequences, *Tepidamorphus gemmatus* CB-27A^T^, *Baulida consociate* 11^T^, *Afifella pfenningi* DSM 17143^T^, and *Amorphus orientalis* YIM D10^T^, with percent similarities of 95.03, 94.53, 94.43, and 94.08, respectively, are phylogenetically closely related. A Roche 454 (FLX titanium) pyrosequencing platform was used to perform the whole genome sequencing of *Lutibaculum baratangense* AMV1^T^, which yielded a sequence of 70,392,118 bp in 163,500 reads, which is a 17-fold coverage of the genome. The GS De Novo Assembler (version 2.8) was used to perform assembly of the raw sequencing reads, which yielded a genome of 4,300,776 bp in 44 contigs with all contigs larger than 1,166 bp and with the largest contig being 670,712 bp. The calculated G+C content of the genome was 66.93 mol%. Rapid annotation using subsystem technology was used to perform annotation of the assembled genome (2), tRNA was predicted by tRNAscan-SE (version 1.23) (3), and rRNA genes were predicted by RNAmmer (version 1.2) (4).

The annotation of *Lutibaculum baratangense* AMV1^T^ consists of a total of 4,198 predicted coding regions, including 56 RNA features. The annotation shows the presence of 48 genes for resistance against antibiotics and toxic compounds, including 15 genes for cobalt, zinc, and cadmium resistance; 4 genes for resistance against arsenic; 5 beta lactamase genes; 6 genes providing resistance against mercury; and 14 genes involved in resistance against copper. Also, the genome consists of 267 genes for membrane transport, including 92 ABC transporters and 76 TRAP transporters. The genome annotation also shows the presence of 22 genes involved in flagellar motility, which may be responsible for the motility of the bacterium. There are also 166 genes involved in stress response, including 70 genes against osmotic shock, 70 against oxidative shock, 16 against heat shock, and 4 against cold shock. The sequencing of the bacterium will aid in better understanding their adaptation to the harsh conditions of the mud volcano.

### Nucleotide sequence accession numbers.

The draft genome sequence of *Lutibaculum baratangense* strain AMV1^T^ has been deposited in DDBJ/EMBL/GenBank under the accession number AWXZ00000000. The version described in this paper is version AWXZ01000000.
